# Novel preclinical model for CDKL5 deficiency disorder

**DOI:** 10.1242/dmm.049094

**Published:** 2022-03-08

**Authors:** Rita J. Serrano, Clara Lee, Alon M. Douek, Jan Kaslin, Robert J. Bryson-Richardson, Tamar E. Sztal

**Affiliations:** 1 School of Biological Sciences, Monash University, Melbourne 3800, Australia; 2 Australian Regenerative Medicine Institute, Monash University, Melbourne 3800, Australia

**Keywords:** CDKL5 deficiency disorder, Motor neurons, Seizure, Locomotion, Microcephaly, Zebrafish

## Abstract

Cyclin-dependent kinase-like-5 (CDKL5) deficiency disorder (CDD) is a severe X-linked neurodegenerative disease characterised by early-onset epileptic seizures, low muscle tone, progressive intellectual disability and severe motor function. CDD affects ∼1 in 60,000 live births, with many patients experiencing a reduced quality of life due to the severity of their neurological symptoms and functional impairment. There are no effective therapies for CDD, with current treatments focusing on improving symptoms rather than addressing the underlying causes of the disorder. Zebrafish offer many unique advantages for high-throughput preclinical evaluation of potential therapies for neurological diseases, including CDD. In particular, the large number of offspring produced, together with the possibilities for *in vivo* imaging and genetic manipulation, allows for the detailed assessment of disease pathogenesis and therapeutic discovery. We have characterised a loss-of-function zebrafish model for CDD, containing a nonsense mutation in *cdkl5*. *cdkl5* mutant zebrafish display defects in neuronal patterning, seizures, microcephaly, and reduced muscle function caused by impaired muscle innervation. This study provides a powerful vertebrate model for investigating CDD disease pathophysiology and allowing high-throughput screening for effective therapies.

This article has an associated First Person interview with the first author of the paper.

## Abstract (precis)

**Summary:** Characterisation of a novel loss-of-function zebrafish model for CDKL5 deficiency disorder, containing a nonsense mutation, demonstrates its utility for investigating disease aetiology and allowing high-throughput screening for potentially effective therapies.

## INTRODUCTION

CDKL5 deficiency disorder (CDD) is a severe neurodegenerative disease caused by mutations in cyclin-dependent kinase-like-5 (*CDKL5*). Patients with CDD often display a heterogeneous array of clinical phenotypes, including early infantile epilepsy, delayed motor function and intellectual disability (Tao et al., 2004). CDKL5 is a member of a highly conserved family of serine-threonine kinases, functioning to regulate cytoskeletal dynamics, synaptic vesicle stability and release, neurite outgrowth, and dendritic spine development (Rusconi et al., 2008). To date, more than 265 variants have been identified in *CDKL5*, with ∼70 of these considered pathogenic (Jakimiec et al., 2020). Many mutations are located within the catalytic domain, and those disrupting catalytic activity lead to a complete loss of kinase function (Bertani et al., 2006; Jakimiec et al., 2020). Owing to the rarity of the disorder (1 in 60,000 live births) very little is known about long-term prognosis; however, many patients die by early adulthood. For most patients, there are daily and ongoing medical challenges associated with managing the severe neurological impairments, as well as the range of progressive and complex clinical comorbidities that can accompany the disease.

There are no effective therapies for CDD, with most treatments focusing on improving symptoms rather than addressing the underlying cause of the disorder. A handful of compounds are currently in clinical trials, including ataluren, currently used to treat Duchenne muscular dystrophy, to promote read-through of CDKL5 nonsense mutations (McDonald et al., 2017), and ganaxolone, to reduce seizure activity (Yawno et al., 2017). The lack of effective therapies is in part due to the complexity of the symptoms exhibited by patients and the limited understanding of CDKL5 function and its associated signal transduction pathways.

Previous studies have shown that Cdkl5 knockout (KO) mice recapitulate many features of CDD, exhibiting severe neuronal impairment leading to deficits in learning, memory and social behaviour (Fuchs et al., 2018; Ren et al., 2019; Tang et al., 2017; Wang et al., 2012). These deficiencies are associated with abnormalities in neurite growth and proliferation, leading to reduced dendritic arborization of cortical neurons and spine density of hippocampal neurons, affecting brain function and visual cortex processing (Amendola et al., 2014; Wang et al., 2012). Significantly, neuronal development was shown to be arrested in Cdkl5 KO mice, suggesting that symptoms may be caused by improper dendritic maturation and formation rather than degeneration of properly formed neural tissues (Chen et al., 2010; Fuchs et al., 2018, 2014; Ren et al., 2019). However, the majority of Cdkl5 KO mice models display only minor motor function deficits, with small alterations observed in their gait suggesting a mild coordination disturbance, and do not exhibit spontaneous seizures as observed in CDD patients (Amendola et al., 2014; Okuda et al., 2018; Wang et al., 2012).

Unlike other vertebrates, the zebrafish model is highly suited to *in vivo* analysis of neurological development and CDD progression. Notably, the optical clarity and power of live imaging, combined with the accessibility of developing zebrafish larvae, provide novel ways for monitoring disease states. Development of both the brain and spinal cord is highly conserved between humans and zebrafish, as well as many signalling pathways involved in their function. In zebrafish, the skeletal muscle makes up ∼80% of the body mass, is functional as early as 20 h post fertilization (hpf) and, after 2 days post fertilization (dpf), the fish swim spontaneously. Muscle contractions controlling early spontaneous motor activity are likely to be directly modulated by embryonic central nervous system activity. At this time, the upper motor neurons in the primary cortex send their projections to the developing spinal cord, and it is only after the fast muscle is formed at ∼52 hpf that the lower motor neurons project to the myotome (D'Elia and Dasen, 2018). Zebrafish are highly amenable to high-throughput preclinical evaluation of potential therapies for severe congenital disorders, including CDD. Measurements of swimming behaviour using automated tracking systems can yield an unbiased, reliable and high-throughput assessment of swimming performance that is reflective of skeletal muscle function and innervation (Sztal et al., 2016).

In zebrafish, *cdkl5* exists as a single gene copy, and bioinformatics analysis has shown that Cdkl5 is highly conserved between zebrafish and its vertebrate orthologues, being 89% identical to the chicken, human and mouse proteins (Katayama et al., 2016). Previous studies have reported that two *cdkl5* transcripts (long and short) are produced during zebrafish development, which differ in the inclusion of exon 16 (Katayama et al., 2016). Both isoforms are expressed during early zebrafish development, specifically in neural tissues, including the brain and eye (Katayama et al., 2016). Mammals are known to have multiple splice variants, with the longest isoform also expressed highly in the human brain (Fichou et al., 2011; Rademacher et al., 2011).

Here, we have characterised a zebrafish model for CDD to study disease progression and pathobiology. We show that *cdkl5^−/−^* zebrafish display reduced body and head size, defects in motor neuron branching and impaired motor function. Using live confocal imaging, we show that *cdkl5^−/−^* zebrafish also display spontaneous seizures, as well as a reduction in total brain volume, revealing a propensity for microcephaly, and in cerebellar volume, which regulates motor function. Together, our results validate this novel disease model that may be exploited for the testing of potential therapeutics for CDD.

## RESULTS

### *cdkl5* is expressed during early zebrafish development

**Fig. 1.**
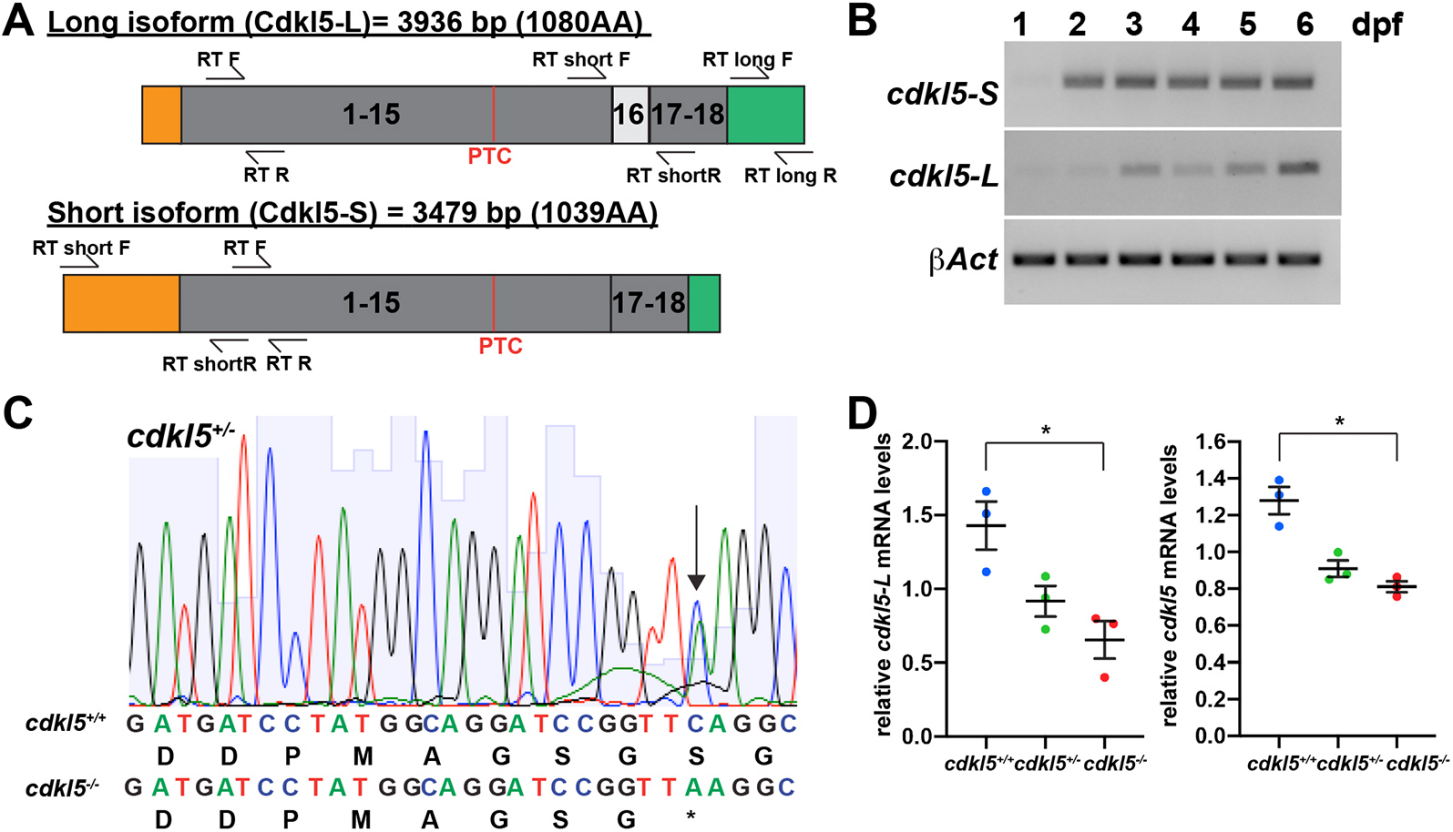
***cdkl5* expression and validation of the *cdkl5^sa21938^* zebrafish mutant strain.** (A) Schematic of the zebrafish Cdkl5 long (Cdkl5-L) and short (Cdkl5-S) protein isoforms showing the location of the nonsense (PTC) mutation and primers used for RT-PCR analyses. The long (3936 bp) and short isoforms (3479 bp) both contain exons 1-15 and exons 17-18 (grey), and differ by the inclusion of exon 16 (white) and the lengths of their 5′ untranslated region (UTR) (orange) and 3′ UTR (green) regions. (B) RT-PCR analyses of *cdkl5-L* and *cdkl5-S* transcripts from 1-6 dpf. * β-Act* was amplified as a positive control. (C) *cdkl5* mutants contain a C>A change in exon 11 of the gene (amino acid 528 out of 1039 and 1080 of the short and long isoforms, respectively), creating a premature termination codon (PTC), as seen in the sequencing chromatograms from *cdkl5^+/−^* fish. (D) qRT-PCR analysis demonstrated that *cdkl5-L* and total *cdkl5* mRNA levels are significantly reduced in *cdkl5^−/−^* embryos compared to *cdkl5^+/+^* siblings at 3 dpf. Data are mean±s.e.m. for three independent experiments (with 20 pooled fish per experiment), *P<0.05 (one-way ANOVA).

Consistent with previous reports (Katayama et al., 2016; Vitorino et al., 2018), both the long and short *cdkl5* isoforms were expressed in the developing embryo (Fig. 1A,B), suggesting that Cdkl5 plays a role in early zebrafish development. To determine the spatial expression of *cdkl5*, we performed *in situ* hybridization, using an antisense *cdkl5* mRNA probe recognising both the long and short isoforms, and found that *cdkl5* is expressed in the brain, muscle and eyes from 1 dpf, in the developing pectoral fins from 2 dpf and in the developing gut tissue (including the kidney) and the notochord (or developing spine) at 3 dpf (Fig. S1A,C). We also used a sense *cdkl5* mRNA probe and observed low and non-specific expression, unlike that observed for the antisense *cdkl5* probe (Fig. S1B). The brain, spinal cord, eyes, muscle and kidney represent tissues that are affected in patients carrying *CDKL5* mutations (Jakimiec et al., 2020), indicating that zebrafish may be a suitable model for studying CDD pathogenesis.

### Loss of *cdkl5* results in impaired muscle movement and neuronal branching

We obtained a *cdkl5* zebrafish mutant containing a C>A change in exon 11 of *cdkl5* (amino acid 528 of both the short and long isoforms) creating a premature termination codon leading to a truncated and non-functional gene product (Fig. 1A). We verified the mutation in this strain by Sanger sequencing, and its location is predicted to affect both the long and short Cdkl5 isoforms (Fig. 1A,C). Using qRT-PCR, we determined that the mutation leads to a significant reduction in *cdkl5* mRNA in *cdkl5^−/−^* fish compared to their wild-type siblings (Fig. 1D).

**Fig. 2.**
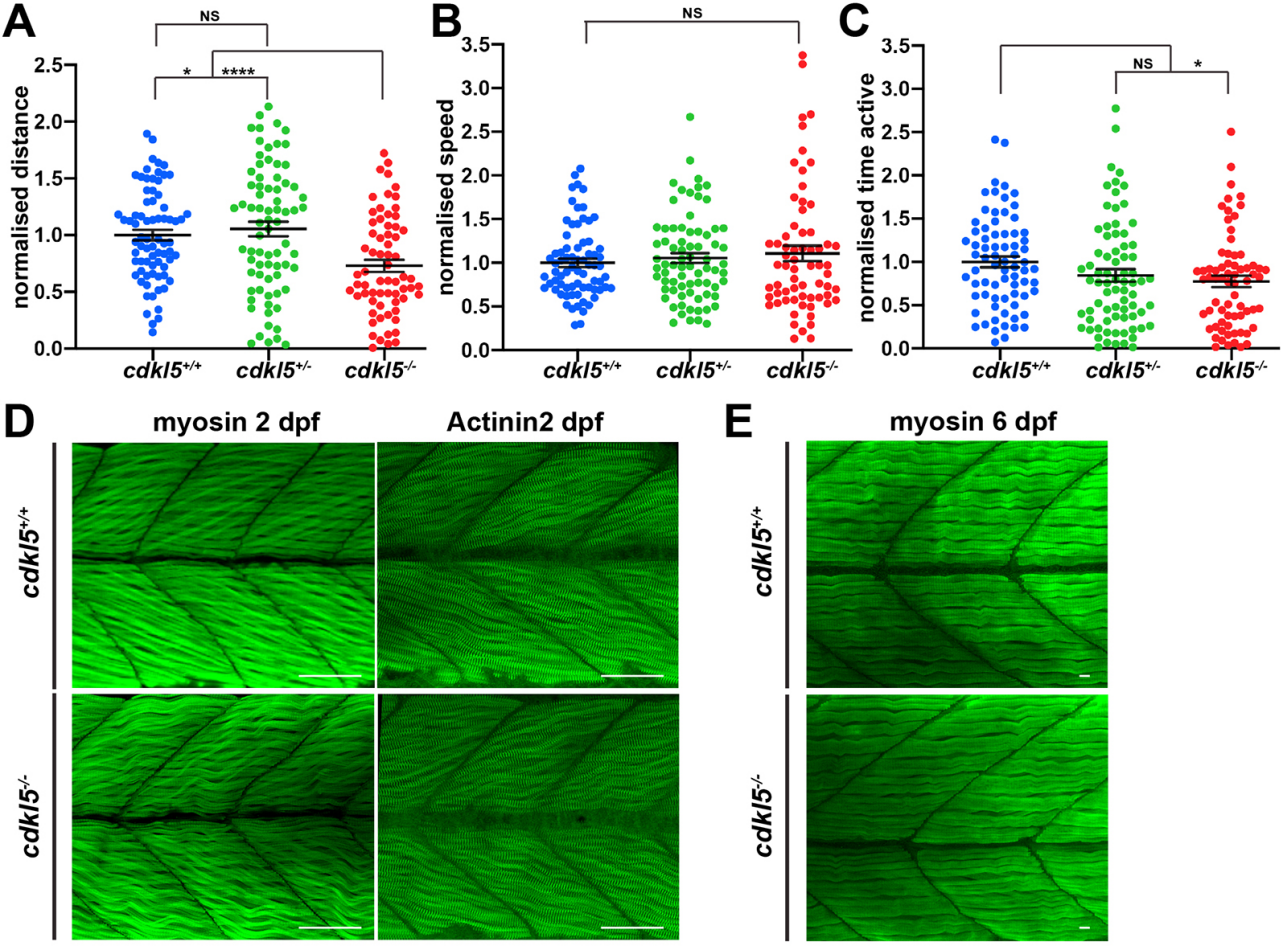
**Motor function and muscle pathology in *cdkl5^−/−^* fish.** (A-C) Quantification of normalized distance travelled (A), speed (B) and time active (C) of *cdkl5^+/+^*, *cdkl5^+/−^* and *cdkl5^−/−^* fish at 6 dpf. Data are mean±s.e.m. for three independent experiments (*n*=38, 21 and 15 for *cdkl5^+/+^*; *n*=17, 39 and 19 for *cdkl5^+/−^*; and *n*=27, 25 and 14 for *cdkl5^−/−^* fish per experiment). *P<0.05; ****P<0.0001; NS, not significant (one-way ANOVA). (D) Maximum intensity projection of a confocal image series of α-myosin and α-Actinin2 antibody staining of *cdkl5^+/+^* and *cdkl5^−/−^* fish at 2 dpf. (E) Maximum intensity projections of confocal images of α-myosin antibody staining of *cdkl5^+/+^* and *cdkl5^−/−^* fish at 6 dpf. Scale bars: 100 µm (D); 10 µm (E).

Patients carrying *CDKL5* mutations display impaired motor function; therefore, we tested the swimming ability of *cdkl5* mutant zebrafish to determine whether motor function was affected. We detected a significant decrease in the normalised distance swum by *cdkl5^−/−^* (0.716±0.0525 mean±s.e.m.) compared to both *cdkl5^+/−^* (1.055±0.0647 mean±s.e.m.) and *cdkl5^+/+^* fish (1.00±0.0469 mean±s.e.m.) (Fig. 2A). Interestingly, there was no significant difference in the swimming speed of *cdkl5^−/−^* fish (1.108±0.0892 mean±s.e.m.) compared to their siblings (1.00±0.0484 for *cdkl5^+/+^* and 1.054±0.0570 for *cdkl5^+/−^*; mean±s.e.m.) (Fig. 2B). We also did not observe any obvious differences in swimming trajectories of *cdkl5^−/−^* fish compared to their wild-type siblings (Fig. S2). However, we did detect a significant decrease in the normalised duration spent swimming by *cdkl5^−/−^* (0.774±0.0663 mean±s.e.m.) compared to *cdkl5^+/+^* fish (1.00±0.0622 mean±s.e.m.), suggesting the reduced distance swum by *cdkl5^−/−^* fish is due to reduced activity (Fig. 2C). We analysed muscle pathology by labelling the trunk muscle for myosin and Actinin2, essential components of muscle fibre. We observed no overt difference in either muscle structure or patterning in *cdkl5^−/−^* compared to *cdkl5^+/+^* fish (Fig. 2D,E) at 2 dpf or 6 dpf.

**Fig. 3.**
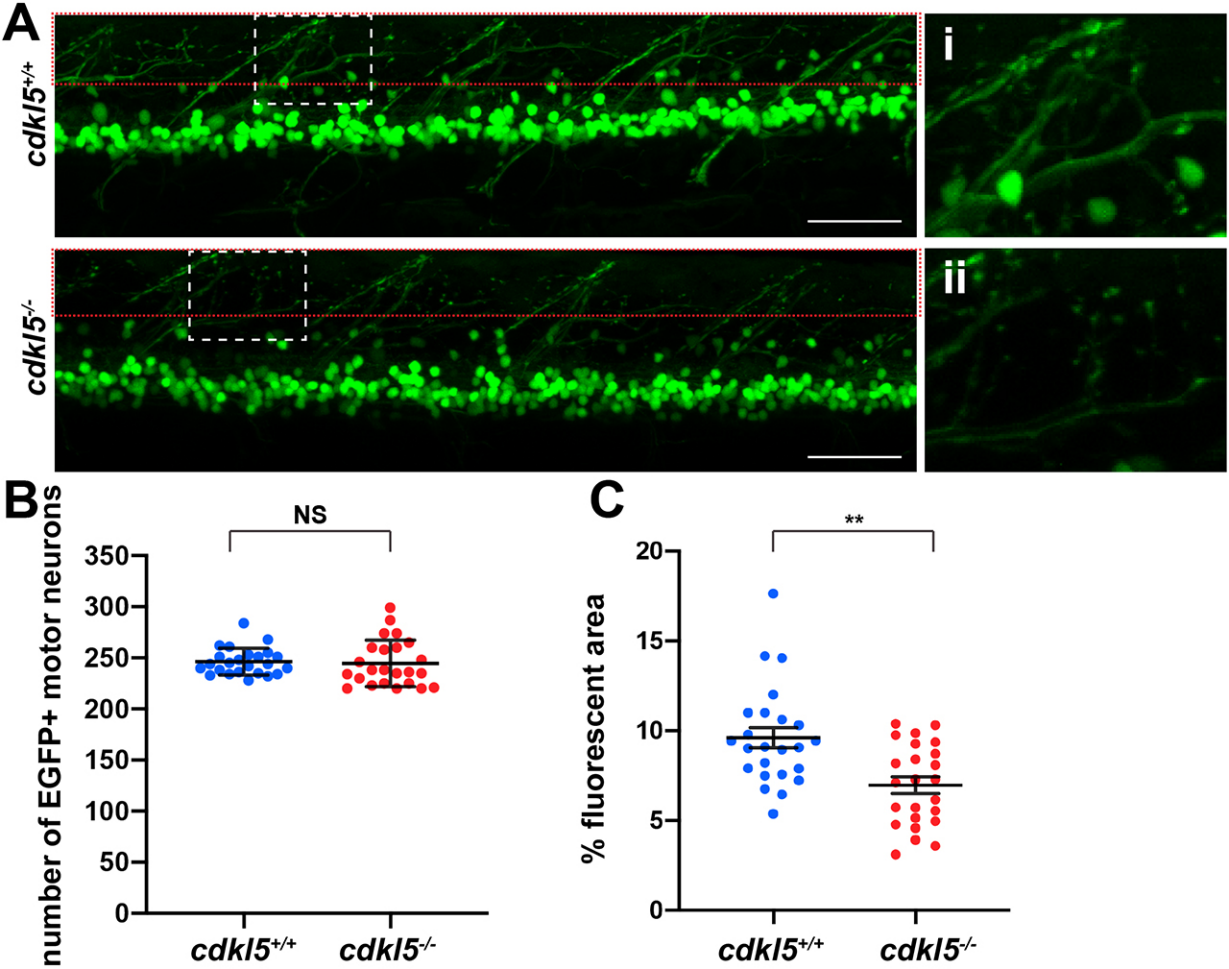
**Assessment of neuronal pathology in *cdkl5^−/−^* fish.** (A) Maximum intensity projection confocal images of EGFP-labelled motor neurons from Tg(*islet1:EGFP*)*; cdkl5^+/+^* (white dashed box zoomed in i) and Tg(*islet1:EGFP*)*; cdkl5^−/−^* (white dashed box zoomed in ii) along the spinal region at 6 dpf. Red dashed boxes indicate the regions quantified in C. Scale bars: 50 µm. (B) Quantification of the number of EGFP^+^ motor neurons at 6 dpf. Data are mean±95% c.i. for three independent experiments [*n*=8, 8 and 8 for Tg(*islet1:EGFP*)*; cdkl5^+/+^*, and *n*=8, 8 and 8 for Tg(*islet1:EGFP*)*; cdkl5^−/−^* fish]. (C) Quantification of the mean percentage area of fluorescence from Tg(*islet1:EGFP*)*; cdkl5^+/+^* and Tg(*islet1:EGFP*)*; cdkl5^−/−^* (within the red dashed box region in A) at 6 dpf. Data are mean±s.e.m. for three independent experiments [*n*=8, 8 and 8 for Tg(*islet1:EGFP*)*; cdkl5^+/+^*, and *n*=8, 8 and 8 for Tg(*islet1: EGFP*)*; cdkl5^−/−^* fish]. **P<0.01; NS, not significant (two-tailed *t*-test).

We then crossed *cdkl5^+/−^* fish to a Tg*(islet1:EGFP)* zebrafish transgenic strain, labelling all of the motor neurons and ventral interneurons with enhanced green fluorescent protein (EGFP) (Higashijima et al., 2000). Using this transgenic approach, we were able to image motor neurons in the live embryo at 6 dpf to determine whether there were significant deviations in cell number and axon formation. Although we recorded no significant difference in the number of cell bodies in Tg(*islet1:EGFP*);*cdkl5^−/−^* (244.6±2.65 mean±s.e.m.) compared to Tg(*islet1:EGFP*);*cdkl5^+/+^* fish (246.2±5.96 mean±s.e.m.) (Fig. 3A,B), we observed a significant reduction in the density of neuronal processes emerging from the spinal cord, revealed by a reduction of fluorescent surface area in Tg(*islet1:EGFP*);*cdkl5^−/−^* (7.004±0.626 mean±s.e.m.) compared to Tg(*islet1:EGFP*);*cdkl5^+/+^* fish (9.600±0.499 mean±s.e.m.) (Fig. 3A,C). This reduced axonal branching likely results in impaired connectivity between the spinal cord and adjacent muscle fibres. However, colocalisation of Synaptic vesicle 2 (SV2) (marking the motor neuron presynaptic receptors) and bungarotoxin (marking the skeletal muscle post-synaptic receptors) did not show any obvious differences in the overall structure of the neuromuscular junctions between *cdkl5^−/−^* and *cdkl5^+/+^* fish at 6 dpf (Fig. S3A).

### *cdkl5* mutant zebrafish show an increased susceptibility to seizures

**Fig. 4.**
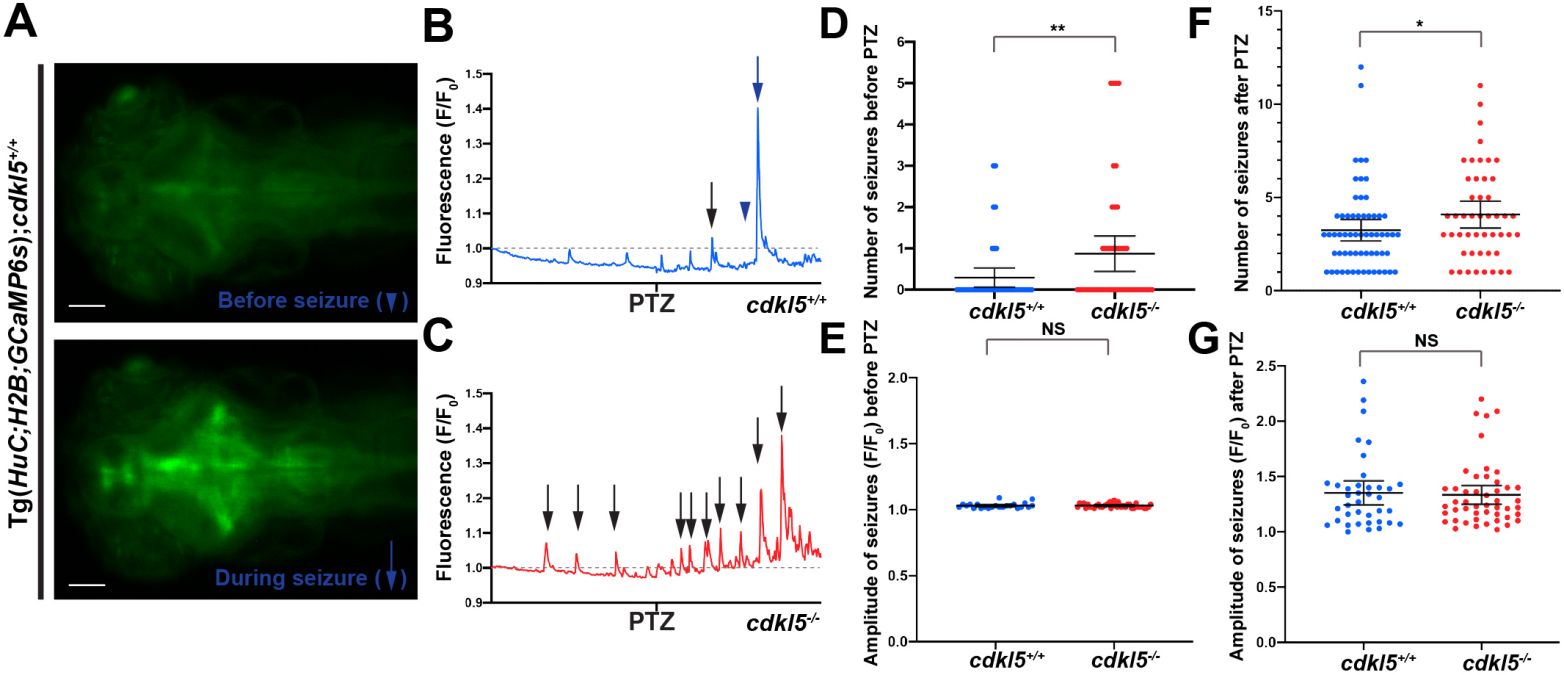
**Analysis of seizures in *cdkl5^−/−^* fish.** (A) Images of the brain region in Tg(*HuC:H2B-GCaMP6s*)*; cdkl5^+/+^* fish before (indicated in B by the blue arrowhead) and during a seizure (indicated in B by the blue arrow) at 6 dpf. Scale bars: 100 µm. (B,C) Representative time trace images of fluorescence (F/F_0_,) in control conditions and after the addition of 20 mM PTZ (at the time indicated) for Tg(*HuC:H2B-GCaMP6s*)*;cdkl5^+/+^* (B) and Tg(*HuC:H2B-GCaMP6s*)*;cdkl5^−/−^* (C) fish. The fluorescence data are integrated over the whole embryonic brain. Seizures (arrows) are represented by peaks in fluorescence that extend above the baseline level (represented by the dotted line). (D,E) Quantification of the number (D) and amplitude (E) of seizures observed before PTZ treatment in Tg(*HuC:H2B-GCaMP6s*)*;cdkl5^+/+^* and Tg(*HuC:H2B-GCaMP6s*)*;cdkl5^−/−^* fish. (F,G) Quantification of the number (F) and amplitude (G) of seizures observed after PTZ treatment in Tg(*HuC:H2B-GCaMP6s*)*;cdkl5^+/+^* and Tg(*HuC:H2B-GCaMP6s*)*;cdkl5^−/−^* fish. Data are mean±95% c.i. for three independent experiments [*n*=9, 12 and 18 for Tg(*HuC:H2B-GCaMP6s*)*;cdkl5^+/+^*, and *n*=19, 13 and 16 for Tg(*HuC:H2B-GCaMP6s*)*;cdkl5^−/−^* fish]. *P<0.05; **P<0.01; NS, not significant (two-tailed *t*-test).

CDD is characterised by early-onset seizures that usually start by 3 months of age and are often refractive to long-term anti-seizure medication. To determine whether *cdkl5* mutants display seizures during early embryonic development, we used a Tg(*HuC:H2B-GCaMP6s*) transgenic strain, in which the genetically encoded calcium indicator GCaMP6s is expressed in mature neurons (Chen et al., 2013). Using live imaging, we observed an increase in the number of spontaneous seizures in Tg(*HuC:H2B-GCaMP6s*);*cdkl5^−/−^* (0.875±0.214 mean±s.e.m.) compared to Tg(*HuC:H2B-GCaMP6s*);*cdkl5^+/+^* fish (0.296±0.115 mean±s.e.m.) (Fig. 4D), associated with spikes in GCaMP6s expression in the brain (Fig. 4A-C). To experimentally induce seizures, we treated *cdkl5^−/−^* and *cdkl5^+/+^* fish with a 20 mM pentylenetetrazole (PTZ) solution, a convulsant that affects GABA and glutamate receptors, which has previously been used as a seizure model in zebrafish (Liu and Baraban, 2019). Following PTZ treatment, we reported a significant increase in the number of seizures exhibited by Tg(*HuC:H2B-GCaMP6s*);*cdkl5^−/−^* (4.083±0.360 mean±s.e.m.) compared to Tg(*HuC:H2B-GCaMP6s*);*cdkl5^+/+^* fish (2.974±0.342 mean±s.e.m.) (Fig. 4F) over a 5 min time period, although the intensity of seizures recorded was not significantly altered both before [1.032±0.003 for Tg(*HuC:H2B-GCaMP6s*);*cdkl5^−/−^* fish and 1.031±0.004 for Tg(*HuC:H2B-GCaMP6s*);*cdkl5^+/+^* fish; mean±s.e.m.] and after PTZ treatment [1.334±0.042 for Tg(*HuC:H2B-GCaMP6s*);*cdkl5^−/−^* fish and 1.351±0.0535 for Tg(*HuC:H2B-GCaMP6s*);*cdkl5^+/+^* fish; mean±s.e.m.] (Fig. 4E,G).

### Morpholino knockdown of Cdkl5 recapitulates phenotypes observed in *cdkl5* mutants

To confirm that a reduction in Cdkl5 causes defects in motor function and muscle innervation, we used a morpholino (MO) targeting the splice donor site of exon 5 (Cdkl5 ex5 MO) to knockdown Cdkl5 levels. To determine an effective dose for knockdown, we injected the MO at 0.5 ng, 1.0 ng or 2.0 ng into wild-type embryos and performed RT-PCR. We were able to detect a number of smaller sized amplicons by RT-PCR only in the 2.0 ng injected samples (Fig. S4A). Based on these observations, we selected 2.0 ng MO to use in further experiments. To confirm that *cdkl5* mRNA levels are reduced in Cdkl5 morphants, we performed RT-PCR analyses at 2 and 6 dpf and found that *cdkl5* mRNA is decreased compared to standard control MO-injected fish (Fig. S4B). We performed locomotion assays at 6 dpf and found a significant decrease in swimming performance in Cdkl5 morphants (0.678±0.0336 mean±s.e.m.) compared to standard control MO-injected fish (1.00±0.0664 mean±s.e.m.) (Fig. S4D). We did not observe any defects in muscle patterning in standard control-MO injected fish or Cdkl5 morphants at 2 and 6 dpf (Fig. S4C), consistent with *cdkl5^−/−^* fish.

To investigate neuronal pathologies in Cdkl5 morphants, we used an anti-α-acetylated tubulin antibody to stain mature neurons, including their axons. We observed no obvious differences in neuronal morphology or projections at 3 dpf between Cdkl5 knockdown and control MO-injected fish (Fig. S4E). However, in Tg*(islet1:EGFP*);Cdkl5 knockdown fish, there appeared to be a reduction in the density of axonal projections (Fig. S4F), recapitulating the pathologies observed in *cdkl5^−/−^* fish (Fig. 3B) and confirming that reduction of Cdkl5 affects zebrafish neuromuscular connectivity.

### *cdkl5* mutants display skeletal abnormalities and microcephaly

**Fig. 5.**
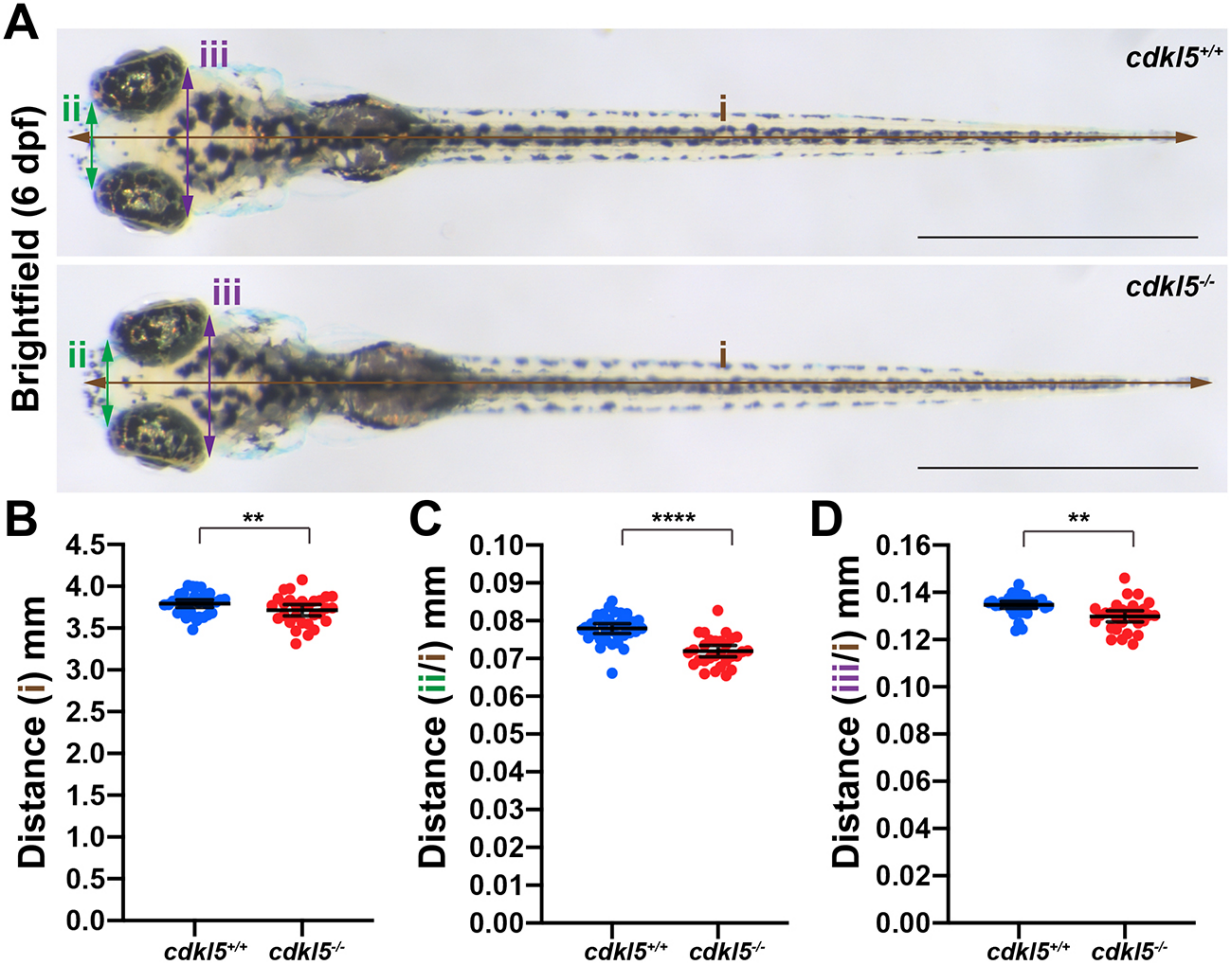
**Analysis of body length and head size in *cdkl5^−/−^* fish.** (A) Bright-field images of *cdkl5^+/+^* and *cdkl5^−/−^* fish at 6 dpf. Lines indicate body length (i, brown), anterior head width (ii, green) and posterior head width (iii, purple). Scale bars: 1 mm. (B-D) Quantification of body length and head width normalised to body length (B) for anterior head width (C) and posterior head width (D) measurements of *cdkl5^+/+^* and *cdkl5^−/−^* fish. Data are mean±95% c.i. for three independent experiments (*n*=11, 13 and 10 for *cdkl5^+/+^*, and *n*=10, 10 and 9 for *cdkl5^−/−^*). **P<0.01, ****P<0.0001 (two-tailed *t*-test).

**Fig. 6.**
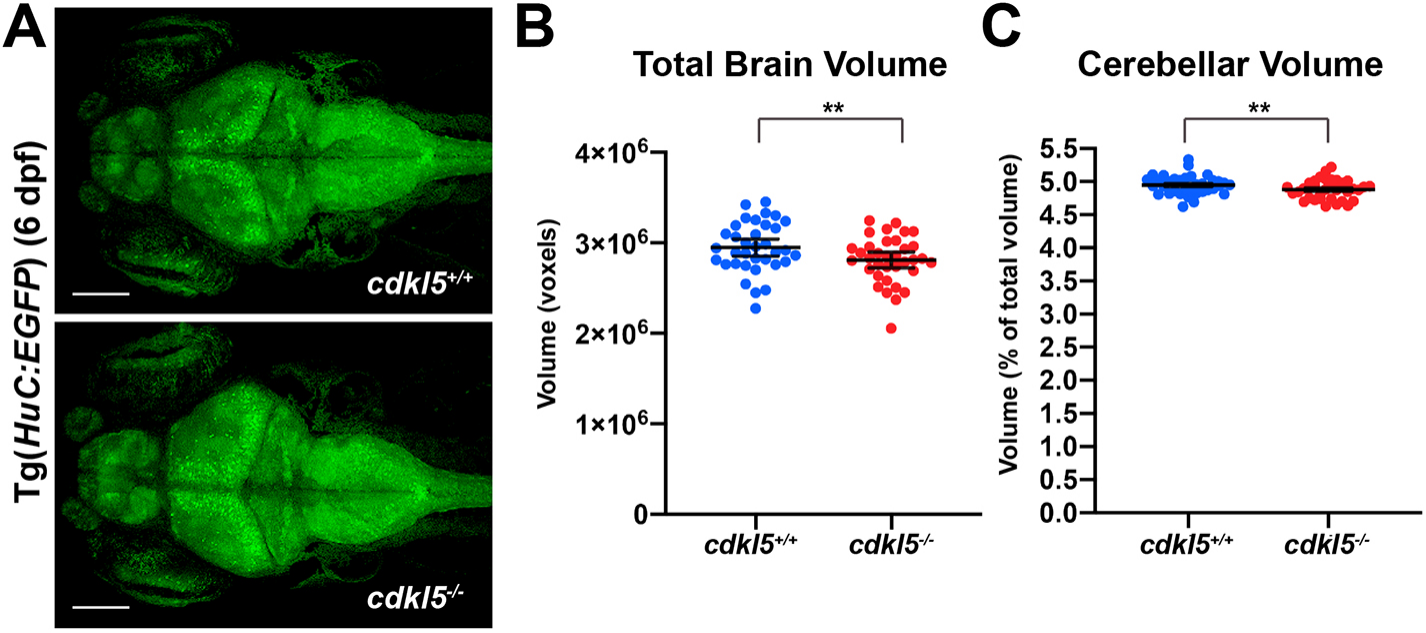
**Brain imaging in *cdkl5^−/−^* fish.** (A) Maximum intensity projections of confocal images from whole brains from Tg(*HuC:EGFP*)*; cdkl5^+/+^* and Tg(*HuC:EGFP*)*; cdkl5^−/−^* fish at 6 dpf. Scale bars: 100 µm. (B,C) Quantification of total brain (B) and cerebellar (C) volume from *cdkl5^+/+^* and *cdkl5^−/−^* fish. Data are mean±s.e.m. for three independent experiments (*n*=12, 12 and 11 for *cdkl5^+/+^*, and *n*=12, 12 and 12 for *cdkl5^−/−^* fish), **P<0.01 (two-tailed *t*-test). 1 voxel=8 µm^3^.

CDD patients have been reported to exhibit low muscle tone and a small head size, with some demonstrating cortical atrophy identified through brain imaging (Yamamoto et al., 2015). To determine whether *cdkl5*^−/−^ fish display reduced head size, we measured body length and head size by taking anterior and posterior width measurements at 6 dpf (Fig. 5A), and normalised the width of the head to the body length. Although we observed a significant decrease in the body length of *cdkl5^−/−^* (3.713 mm±0.0330 mean±s.e.m.) compared to *cdkl5^+/+^* fish (3.793 mm±0.022 mean±s.e.m.) (Fig. 5A,B), there was a significant reduction in the width of the head in *cdkl5^−/−^* (anterior head width, 0.0719 mm±0.001, and posterior head width, 0.130 mm±0.001; mean±s.e.m.) compared to *cdkl5^+/+^* fish (anterior head width, 0.0780 mm±0.001, and posterior head width, 0.135 mm±0.001; mean±s.e.m.) at 6 dpf (Fig. 5A,C,D), recapitulating microcephaly phenotypes observed in CDD patients. We also measured brain volume in *cdkl5^−/−^* zebrafish at 6 dpf using live confocal microscopy and a pan-neuronal fluorescent reporter strain [Tg(*HuC:EGFP*)] (Park et al., 2000). We recorded a 5% reduction (*P*<0.01) in total brain volume in Tg(*HuC:EGFP*)*;cdkl5^−/−^* (2,811,034 voxels±30,820 mean±s.e.m.) compared to Tg(*HuC:EGFP*)*;cdkl5^+/+^* fish (2,944,860 voxels ±26,472 mean±s.e.m.) (Fig. 6A,B), in line with our previous observations (Fig. 5C,D). Additionally, we measured the total volume of the cerebellum (comprising the eminentia granularis, valvula cerebelli, corpus cerebelli and cerebellar neuropil) and recorded a 1.4% reduction (*P*<0.01) in the cerebellar volume of Tg(*HuC:EGFP*)*;cdkl5^−/−^* (4.880±0.0153 mean±s.e.m.) compared to Tg(*HuC:EGFP*)*;cdkl5^+/+^* fish (4.948±0.0162 mean±s.e.m.) (Fig. 6C). We stained the neuronal tissues with an antibody against glutamate synthetase (GS) to mark radial glia and Müller glia in the brain and retina, respectively, and DAPI, and observed no obvious changes in retinal morphology or brain architecture in *cdkl5^−/−^* compared to *cdkl5^+/+^* fish (Fig. S3B,C).

**Fig. 7.**
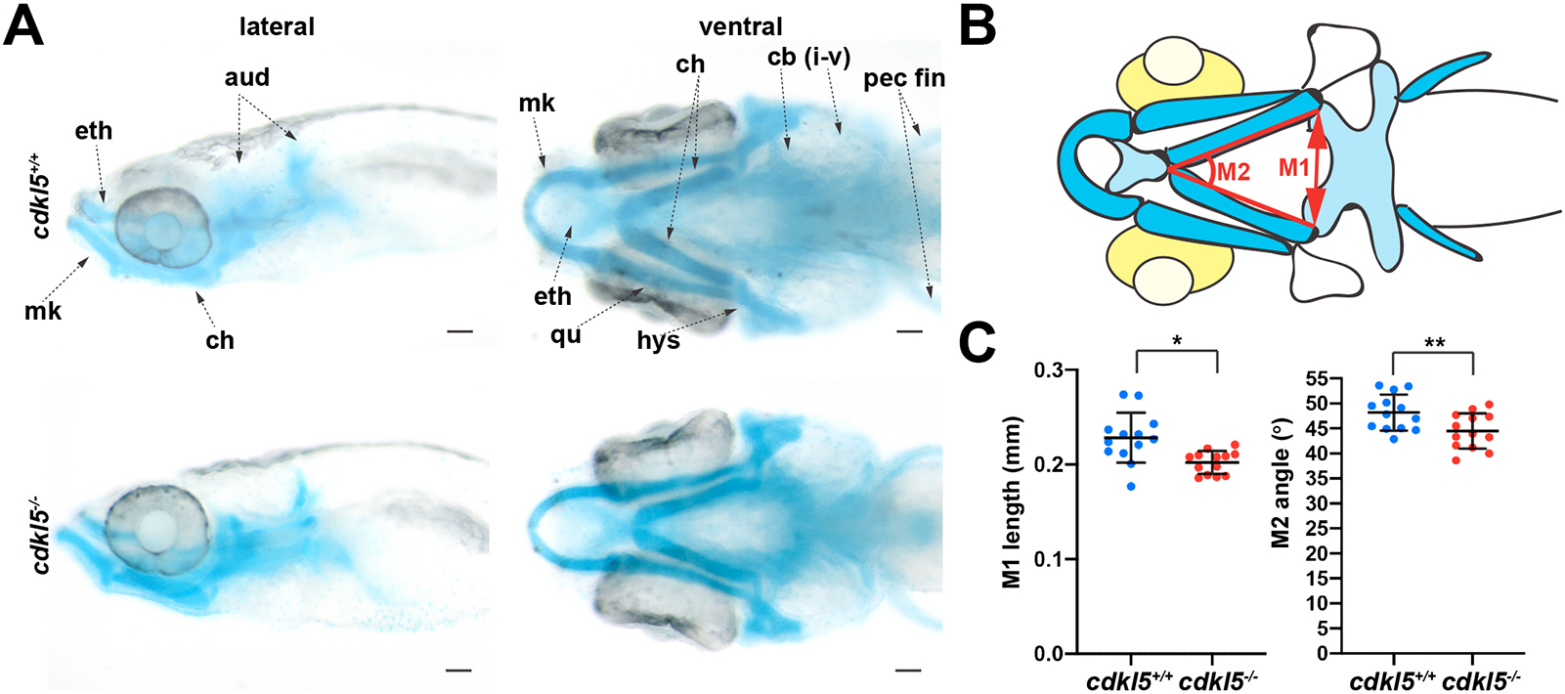
**Alcian Blue staining of craniofacial cartilage structures in *cdkl5^−/−^* fish.** (A) Dorsal and ventral images of Alcian Blue staining in *cdkl5^+/+^* and *cdkl5^−/−^* fish at 7 dpf. aud, auditory capsule; cb, ceratobranchials or gill arch derivatives; ch, ceratohyal; eth, ethmoid plate; hys, hyosymplectic; mk, Meckel's cartilage; pec fin, pectoral fins; qu, quadrate. Scale bars: 100 µm. (B) Schematic of Alcian Blue staining in zebrafish heads at 7 dpf, showing locations of head measurements. (C) Quantification of the length (M1) and intersecting angle (M2) of the ceratohyal cartilage structures in *cdkl5^+/+^* and *cdkl5^−/−^* fish at 7 dpf. Data are mean±s.d. (*n*=13 fish per genotype), *P<0.05, **P<0.01 (two-tailed *t*-test).

To further examine the presence of a microcephaly phenotype, we examined the morphology of cranial cartilage in *cdkl5^−/−^* and *cdkl5^+/+^* fish. The cranial cartilage of the zebrafish is the first skeletal structure present and forms at ∼3 dpf. As zebrafish grow, the bones continue to ossify, with the first osteoblasts surrounding the cartilage and forming bone matrix by 7 dpf (Bergen et al., 2019). We stained the developing cartilage using an Alcian Blue stain (Fig. 7A) and found a significant decrease in both the distance and angle between the two ceratohyal cartilage elements in *cdkl5^−/−^* (distance, 0.202 mm±0.012, and angle, 44.47±0.9822; mean±s.d.) compared to *cdkl5^+/+^* fish (distance, 0.228 mm±0.026, and angle, 48.18±0.996; mean±s.d) at 7 dpf (Fig. 7B,C). This provides further evidence that *cdkl5^−/−^* fish display generalised microcephaly.

## DISCUSSION

To date, no effective therapies have been identified for CDD, with current treatments focusing on the alleviation of symptoms rather than addressing the underlying biology of the disease. A vertebrate animal model that can reliably be used to identify effective therapies is therefore needed. Here, we describe the characterisation of a novel zebrafish model for CDD. *cdkl5* mutant zebrafish display seizure activity, reduced muscle function, impaired neuronal formation and microcephaly, consistent with phenotypes observed in CDD patients.

So far, more than 265 variants have been reported in *CDKL5*, with mutations distributed along the length of the protein (Jakimiec et al., 2020). Approximately 27% of these are considered pathogenic, with many located in the catalytic domain (Jakimiec et al., 2020). Studies have shown that patients with mutations within the catalytic domain and frameshift mutations located at the end of the C-terminal region have more severe motor impairment, refractory (or non-drug responsive) epilepsy or microcephaly (Russo et al., 2009). Milder forms of the disease were caused by mutations in the ATP-binding region or nonsense mutations in the C-terminal regions, with patients possessing better hand coordination and the ability to walk unaided (Bahi-Buisson and Bienvenu, 2012; Olson et al., 2019). The nonsense mutation in *cdkl5^−/−^* zebrafish sits 240 amino acids downstream of the catalytic domain and activates nonsense-mediated decay pathways, causing degradation of the resultant mutant mRNA. Homozygous *cdkl5^−/−^* zebrafish display a 50% reduction in *cdkl5* mRNA, which is comparable to *Cdkl5* mRNA levels detected in Cdkl5 KO female mice models (Fuchs et al., 2018) and heterozygous *CDKL5* mutations observed in patients (Bahi-Buisson et al., 2012). Therefore, we expect that phenotypes observed in *cdkl5^−/−^* zebrafish will be comparable in severity to those contained within the catalytic domain.

Despite the extensive characterisation of neurological phenotypes, there is still not a clear correlation between the type of mutation and phenotypic severity; however, delayed neurodevelopment appears to be a hallmark feature of CDD (Bahi-Buisson and Bienvenu, 2012). CDKL5 belongs to the serine-threonine kinase family and is an important factor influencing neuronal functions (Kilstrup-Nielsen et al., 2012). Interestingly, the levels of CDKL5 vary at different stages of development, with lowest expression in the prenatal stages and increasing throughout postnatal development (Bahi-Buisson and Bienvenu, 2012; Rusconi et al., 2008). The changes in the levels of CDKL5 are consistent with a role in the process of neuronal formation, growth and, in particular, in dendrite development and bifurcation (Jakimiec et al., 2020). Similarly, we found that *cdkl5* mRNA expression increases as development proceeds and reported that neuromuscular connectivity is impaired in *cdkl5^−/−^* zebrafish during early embryonic stages. Interestingly, we observed no significant difference in the number of motor neurons along the spinal cord but recorded a marked decrease in the density of motor neuron processes emerging from the spinal cord in *cdkl5* mutants at 6 dpf, consistent with phenotypes observed in CDD patients and Cdkl5 KO mice (Fuchs et al., 2018; Pizzo et al., 2016). At this age, *cdkl5* mutant zebrafish displayed reduced swimming; however, we did not detect any defects in muscle formation or patterning during early muscle development. Moreover, we did not detect any clear differences in SV2 and bungarotoxin colocalization, suggesting that synapses at neuromuscular junctions are intact in *cdkl5^−/−^* fish. However, further high-resolution imaging may be needed to reveal more subtle defects in *cdkl5^−/−^* fish, accounting for reduced skeletal muscle innervation. Defects in motor neuron axon length and branching have been shown in zebrafish models of motor neuron disease to correlate with shorter swimming distances (Robinson et al., 2019). This supports the idea that the defects in swimming performance of *cdkl5* mutant zebrafish may be caused by impaired motor neuron branching.

Epileptic seizures are usually the earliest symptom of CDD, and in ∼96% of patients they occur within the first 6 months of age (Mangatt et al., 2016). Epileptic seizures often manifest in different forms, with patients experiencing clonic or convulsive seizures, including twisting and jerking motions and tonic or paralysing seizures, resulting in a loss of movement (Demarest et al., 2019). These seizures have often been difficult to detect in an animal disease model and have proven resistant to drug therapies (Frullanti et al., 2019). Until recently (Liao et al., 2020 preprint), none of the published Cdkl5 KO mice exhibited signs of seizures or severe motor dysfunction, which may be due to upregulation of functionally redundant proteins to compensate for the loss of Cdkl5 (Amendola et al., 2014; Okuda et al., 2018; Wang et al., 2012). In contrast, we observed spontaneous seizures in *cdkl5* mutants and recorded a significant increase in seizures in *cdkl5^−/−^* compared to *cdkl5^+/+^* fish following PTZ treatment. This is in line with previous studies in *Cdkl5^R59X^* mice that reported increased seizure activity following injections of subthreshold doses of PTZ (Yennawar et al., 2019). Interestingly, both *cdkl5^−/−^* fish and *Cdkl5^R59X^* mice did not show significant differences in the overall severity and duration of seizures recorded (Yennawar et al., 2019), although it is not known whether seizure activity can change over time in CDD patients (Jakimiec et al., 2020). There was a large variability in swimming trajectories noted for all genotypes tested, and we did not observe an obvious difference in the swimming paths of *cdkl5*^−/−^ fish compared to wild-type siblings over the 10-min locomotion assays. However, it is possible that if fish were allowed to swim in a larger area or were recorded for a longer period of time, jerky swimming motions indicative of seizure-like activity may become more apparent.

We have also shown that *cdkl5^−/−^* zebrafish display reduced head size and a reduction in brain volume akin to CDD patients (Bahi-Buisson et al., 2012). In CDD patients, head circumference at birth is normal in ∼93% of cases; however, in the first few years of life, head circumference is reduced compared to unaffected children of the same age (Frullanti et al., 2019). In severe cases, reduced head size and microcephaly is often accompanied by encephalopathy, causing profound mental retardation (Fehr et al., 2016). Examination of *cdkl5^−/−^* fish revealed a significant reduction in body length compared to *cdkl5^+/+^* siblings, suggesting that growth is impeded during early embryonic development, a phenotype that was also observed in Cdkl5 KO mice (Fuchs et al., 2018). Interestingly, when accounting for the decrease in body length, head size still decreased significantly in *cdkl5^−/−^* fish. We also assessed overall brain and retinal architecture, and there appeared to be no clear morphological evidence of developmental delay on a cellular level between *cdkl5^−/−^* fish and their wild-type siblings to account for the observed differences. Examination of the cerebellum revealed a clear reduction in cerebellar volume in *cdkl5^−/−^* fish compared to their wild-type siblings. The cerebellum is the major brain region responsible for controlling voluntary movements, including motor function, balance and coordination, and defects in cerebellum formation may contribute to impaired motor function in *cdkl5* mutants. Indeed, with *in vivo* imaging, we are limited in our ability to determine whether this difference results from a reduction in brain cell number. However, our analysis of spinal motor neurons suggests that cranial neuronal projections may also be reduced, accounting for the reduced brain volumes recorded in *cdkl5^−/−^* fish.

In conclusion, our study has validated a novel zebrafish model for CDD that will provide mechanistic insight into CDD biology and pathogenesis. The ability to record and quantify seizures live in a whole-animal model is a huge strength of the zebrafish system over other models available. This provides an excellent system in which to perform high-throughput chemical screening to identify effective therapies for CDD that can be directly translated to patients.

## MATERIALS AND METHODS

### Ethics approval

Fish maintenance and handling was carried out as per the standard operating procedures approved by the Monash Animal Ethics Committee under breeding colony license MARP/2015/004/BC.

### Zebrafish maintenance, morpholino injections and genotyping

Zebrafish were maintained according to standard protocols (Westerfield, 2007). Zebrafish strains used were Tg(*islet1:EGFP*) (Higashijima et al., 2000), Tg(*HuC:EGFP*) (Park et al., 2000), Tg(*HuC:H2B-GCaMP6s*) (Chen et al., 2013) and an N-ethyl-N-nitrosourea-generated *cdkl5* mutant line (sa21938) obtained from the Zebrafish International Resource Centre. For *cdkl5* genotyping, Kompetitive allele-specific PCR technology (Geneworks) was used. For MO injections, the Cdkl5 ex5 (5′-AGATATAAACACTGTCATACCTCTG-3′) and standard control (5′-CCTCTTACCTCAGTTACAATTTATA-3′) MOs (GeneTools) were diluted in distilled water and co-injected with Cascade Blue-labelled dextran (Molecular Probes) into one- to two-cell wild-type (Tübingen, TU strain) embryos. MO concentrations were calibrated according to Yuan and Sun (2009) at the indicated concentrations (0.125 mM, 0.25 mM and 0.5 mM for the Cdkl5 ex5 MO corresponding to 0.5, 1.0 or 2.0 ng; and 0.5 mM for the standard control MO corresponding to 2.0 ng). At 1 dpf, the embryos were sorted for Cascade Blue labelling.

### Whole-mount *in situ* hybridisation

Whole-mount *in situ* hybridization was carried out as described previously (Ruparelia et al., 2012). Probes were constructed using *cdkl5* specific primers (F, 5′-GGATCTAAACGCAGCTCCTG-3′, and R, 5′-GGATCTAAACGCAGCTCCTG-3′).

### cDNA synthesis and reverse transcription-PCR

For the qRT-PCR experiment, head and tails were separated from 3 dpf zebrafish, the progeny of a *cdkl5^+/−^* incross, for three independent biological replicates. The tails were used for genotyping and the heads were used for RNA extraction, with 20 embryos per genotype per replicate. For MO experiments, whole embryos were analysed at either 2 or 6 dpf. Total RNA was extracted using TRIzol reagent (Invitrogen Life Technologies). cDNA was synthesised from 1 μg of each RNA sample in a 20 μl reaction using a Protoscript first strand cDNA synthesis kit (New England Biosciences), oligo(dT)20 and Random Primer Mix primers following the supplier's instructions. Primers used for RT-PCR were *cdkl5RTF*, 5′-CTCCGTACGCTCAAACAAGAC-3′, and *cdkl5RTR*, 5′-TGAGCTCTCCCAAAATACACC-3′; *cdkl5short*F, 5′-CGCTTTTATGCTCAATGCAC-3′, and *cdkl5shortR*, 5′-AGCATCTTCAGCTCCCGTAA-3′; *cdkl5longF*, 5′-GCCAGAAGGTGGCAATAGTT-3′, and *cdkl5longR*, 5′-CCCGTCCCTAAGTTTCCATA-3′; *cdkl5MOF*, 5′-CTCCGTACGCTCAAACAAGAC-3′, and *cdkl5MOR*, 5′-TGAGCTCTCCCAAAATACACC-3′; *βActF*, 5′-GCATTGCTGACCGTATGCAG-3′, and *βActR*, 5′-GATCCACATCTGCTGGAAGGTGG-3′; and *ef1αF*, 5′-CTGGAGGCCAGCTCAAACAT-3′, and *ef1αR*, 5′-ATCAAGAAGAGTAGTACCGCTAGCATTAC-3′.

### Locomotion assays

Locomotion assays were performed on 6 dpf zebrafish as per Sztal et al. (2016). An inactivity threshold of 1 mm/s, detection threshold of 25 mm/s and maximum burst threshold of 30 mm/s were used. The total distance swum above inactivity threshold and below maximum burst threshold in a 10-min period was extracted using ZebraLab software (Viewpoint Life Sciences).

### Antibody staining and confocal microscopy

Immunofluorescence was performed on 2, 3 and 6 dpf zebrafish as per Sztal et al. (2015). Antibodies used were anti-α-Actinin2 (Sigma-Aldrich, clone A7811, 1:200); anti-α-myosin heavy chain antibody [Developmental Studies Hybridoma Bank (DSHB), clone A4.1025, 1:10]; anti-α-acetylated tubulin antibody (Sigma-Aldrich, T6793, 1:1000); anti-α-bungarotoxin (Biotium, 00010, 1:500); anti-synaptic vesicle glycoprotein 2A (DSHB, 1:10); anti-α-glutamine synthetase (clone GS-6, Merck MAB302, 1:50); and an Alexa Fluor-labelled-488 secondary antibody (Molecular Probes, 1:200). DAPI (Thermo Fisher Scientific, 1:1000) staining was performed on 6 dpf zebrafish. Imaging was carried out using an LSM 710 confocal microscope (Zeiss) equipped with a 20×1.0 numerical aperture water dipping objective and a 488 nm or 561 nm laser.

### Pentylenetetrazole treatment and seizure analysis

*cdkl5^+/−^* fish were crossed to the Tg(*HuC:H2B-GCaMP6s*) line and raised to adulthood. Tg(*HuC:H2B-GCaMP6s*)*;cdkl5^+/−^* fish were crossed to *cdkl5^+/−^* fish, and their progeny were raised in embryo medium (5 mM NaCl, 0.17 mM KCl, 0.33 mM CaCl_2_ and 0.33 mM MgSO_4_ in water) containing 200 µM N-phenylthiourea (PTU, Sigma-Aldrich) from 12 hpf to suppress melanocyte formation, changing medium every 24 h. Embryos were sorted for EGFP fluorescence at 2 dpf. At 4 dpf, fish were anaesthetised using 0.0016% tricaine methanesulfonate (Sigma-Aldrich) in embryo medium, and DNA was extracted from a small tail clipping and used to genotype embryos (as described above).

At 6 dpf, embryos were again anaesthetised and immobilised in 0.5% low-melting-point agarose in embryo medium. Embryos were first imaged for 5 min in standard embryo medium, after which point PTZ (Sigma-Aldrich, P6500) dissolved in standard embryo medium (to a final concentration of 20 mM, Afrikanova et al., 2013) was added, and embryos were incubated for 3 min. Embryos in PTZ solution were then imaged for a further 5 min. Images were captured every 0.5 s using a Zeiss Axio Z1 Imager compound fluorescent microscope with a Zeiss EC Plan-NEOFLUAR 5×0.16 NA objective. Images of each timepoint for individual embryos were collated into a separate stack in Fiji for both standard embryo medium (control) and PTZ treatments. A region of interest around the brain was selected for each fish, and the mean fluorescence intensity for each timepoint (F) was calculated. The baseline fluorescence level (F_0_) was calculated from the first image taken for each fish in standard embryo medium. The final fluorescence values (F/F_0_) were normalised by dividing the mean fluorescence intensity (F) by the baseline value (F_0_).

### Alcian Blue staining

*cdkl5^+/−^* fish were incrossed and their progeny were raised in embryo medium containing 200 µM PTU (Sigma-Aldrich) from 12 h to suppress melanocyte formation, changing the medium every 24 h. At 7 dpf, embryos were fixed in 4% paraformaldehyde and left overnight in methanol at 4°C. Embryos were rinsed in acid alcohol (70% EtOH:1% HCl) and stained overnight at room temperature in 0.1% Alcian Blue (80% EtOH:20% Acetic Acid). Embryos were washed for 6 h in acid alcohol and then rinsed in PBS plus Tween 20 before imaging.

### Brain image registration and analysis

*cdkl5^+/−^* fish were crossed to the Tg(*HuC:EGFP*) line and raised to adulthood. Tg(*HuC:EGFP);cdkl5^+/−^* fish were crossed to *cdkl5^+/−^* fish, and their progeny were raised in embryo medium containing 200 µM PTU (Sigma-Aldrich) from 12 hpf to suppress melanocyte formation, changing the medium every 24 h. Embryos were sorted for EGFP fluorescence at 2 dpf. At 4 dpf, fish were anaesthetised using 0.0016% tricaine methanesulfonate in embryo medium, and DNA was extracted from a small tail clipping and used to genotype embryos (as described above). At 6 dpf, embryos were again anaesthetised and set in 1% low-melting-point agarose in embryo medium containing tricaine in 0.8 mm fluorinated ethylene propylene tubing (Bola). Images were taken using a Thorlabs confocal microscope with an Olympus 20× water dipping 1.0 NA objective (pinhole 25 µm, 2.005 µm/pixel, step size=1 µm, averaging=16 frames). Brain image registration and total brain and cerebellar volume analysis was performed as per Dark et al. (2020) and Gupta et al. (2018), combining the results from all cerebellar regions to determine total cerebellar volume.

### Motor neuron analyses

*cdkl5^+/−^* fish were crossed to the Tg(*islet1:EGFP*) line and raised to adulthood. Tg(*islet1:EGFP*)*;cdkl5^+/−^* fish were crossed to *cdkl5^+/−^* fish, and their progeny were sorted for EGFP fluorescence at 2 dpf. At 3 dpf, fish were anaesthetised using 0.0016% tricaine methanesulfonate in embryo medium, and DNA was extracted from a small tail clipping and used to genotype embryos (as described above). At 6 dpf, embryos were again anaesthetised and set in 1% low-melting-point agarose in embryo medium containing tricaine, and imaged using an LSM 710 confocal microscope (Zeiss) equipped with a 20× water dipping 1.0 NA objective and a 488 nm laser. Images were taken from comparable regions along the spinal cord for each fish. The total number of EGFP^+^ motor neuron cell bodies in Tg(*islet1:EGFP*) zebrafish were counted from a maximum projection image using the cell counter function in Fiji. The axonal density was determined by quantifying the surface of fluorescence above background. Each maximum projection Tg(*islet1:EGFP*) zebrafish image was cropped to remove any cell bodies. The background was normalised across images, and an upper threshold level of 64,000 and lower threshold level of 9000 was used for each image. The images were converted to a mask image and the percentage area of EGFP^+^ neuronal projections was determined using Fiji.

### Statistics

For all experiments, the investigators were blinded to genotype during data capture and once the analyses were completed the genotypes of the fish were resolved. For swimming analyses, all values were normalised to the average value of *cdkl5^+/+^* siblings. All statistical analyses were performed in GraphPad Prism 7 using a one-way ANOVA for qRT-PCR (Fig. 1D) and *cdkl5* mutant swimming assays (Fig. 2A,B), a two-tailed *t*-test for motor neuron analyses (Fig. 3C), seizure analyses (Fig. 4D-F**)**, growth measurements (Fig. 5B,C), brain measurements (Fig. 6B,C), Alcian Blue staining (Fig. 7C) and Cdkl5 MO swimming assays (Fig. S4D). Data in Fig. 2C, Fig. 3B and Fig. 5D did not pass normality testing and so a Kruskal–Wallis test was performed to determine significance. All other data were tested for normal distribution and passed using D'Agnostino and Perron's test for Gaussian distribution. For brain measurements (Fig. 6), we performed an outlier test using the robust regression and outlier detection (ROUT) method and identified one outlier (*cdkl5^+/+^* embryo 4 sample from replicate 4), which was excluded from the analysis.

## Supplementary Material

Supplementary information
