# Supplementary material for: Novel preclinical model for CDKL5 deficiency disorder
Source: Dis Model Mech. 2022 Mar 8;15(3):dmm049094. doi: 10.1242/dmm.049094 (PMC8922025; doi:10.1242/dmm.049094)
Supplement: Supplementary information [file dmm-15-049094-s1.pdf]

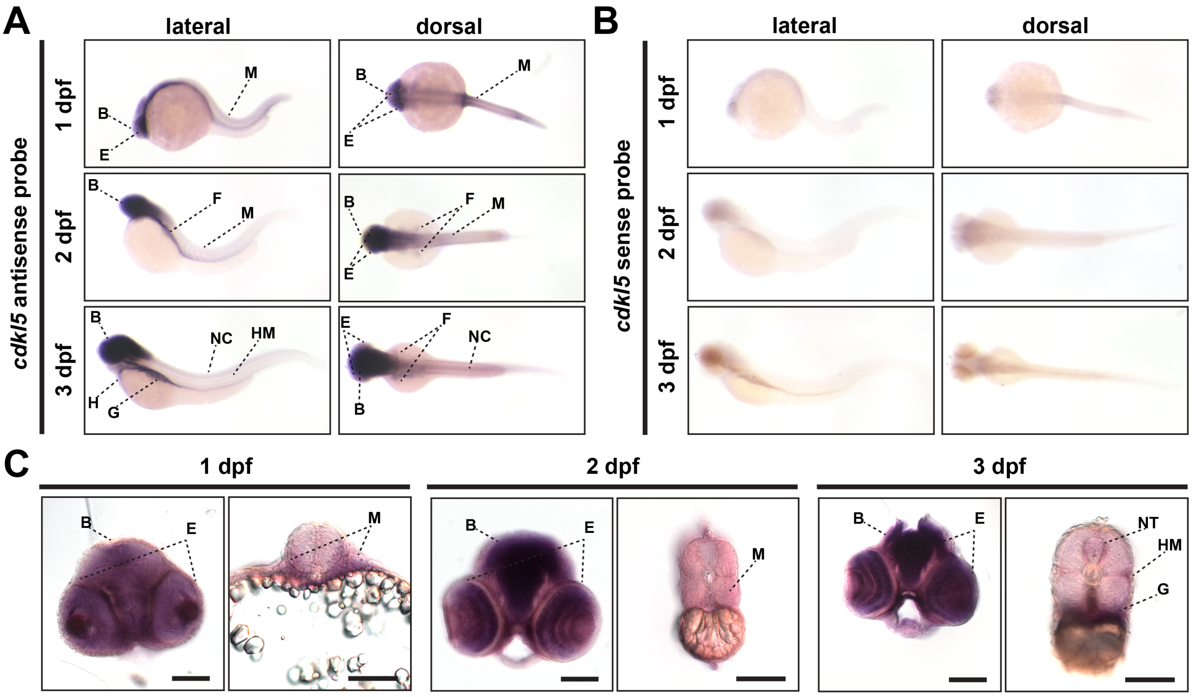

Fig. S1. *In situ* hybridisation of *cdkl5* expression during early zebrafish development. A&C) Using a *cdkl5* antisense probe, we detected *cdkl5* expression in A) whole-mount embryos and C) tissue sections in the brain, eyes, pectoral fins, and skeletal muscle along the trunk at 1 dpf and 2 dpf. At 3 dpf, *cdkl5* is detected in the brain, heart, eyes, developing gut, notochord and horizontal myosepta. Scale bar = 100µm. B) Low level background staining was observed using a *cdkl5* sense probe. (B=brain, E=eye, F=pectoral fins, G=gut primordium, H=heart, HM=horizontal myosepta, K=kidney, M=muscle, NC=notochord).

Disease Models & Mechanisms • Supplementary information

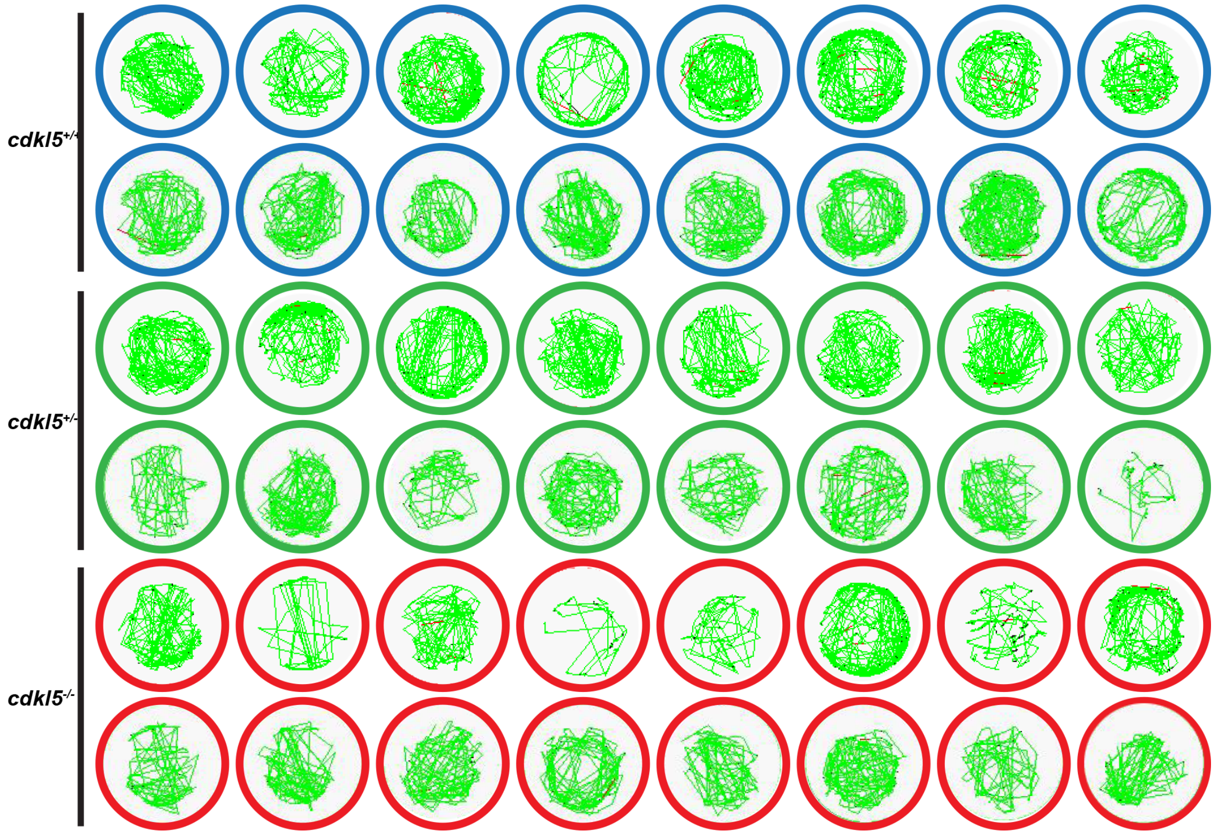

Fig. S2. Swimming trajectories of *cdkl5*$^{-/-}$ fish. Representative images of swimming trajectories of *cdkl5*$^{+/+}$, *cdkl5*$^{+/-}$, and *cdkl5*$^{-/-}$ fish for 10-min locomotion assays at 6 dpf. Green depicts activity within the detection threshold and red depicts movement above the maximum burst threshold.

Disease Models & Mechanisms • Supplementary information

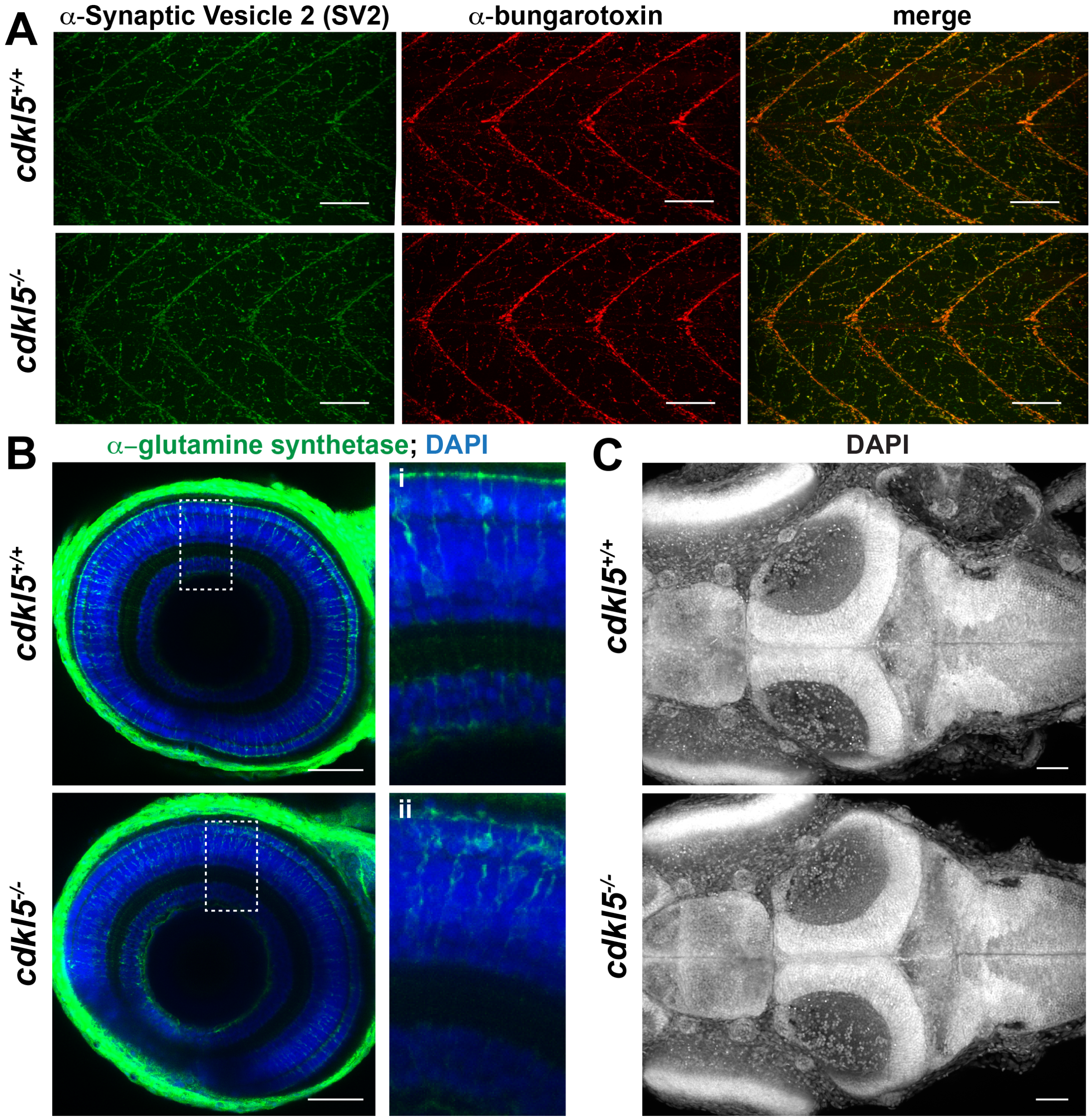

Fig. S3. Assessment of neurological phenotypes in *cdkl5*$^{-/-}$ fish at 6 dpf. Maximum intensity projections of confocal images of A) Synaptic Vesicle 2 antibody staining and α-bungarotoxin staining of skeletal muscle, B) DAPI and α-glutamine synthetase antibody staining of eyes and C) DAPI staining on brain tissue in whole-mount *cdkl5*$^{+/+}$ and *cdkl5*$^{-/-}$ fish. Scale bar for A&B) = 100μm and for C) = 50μm.

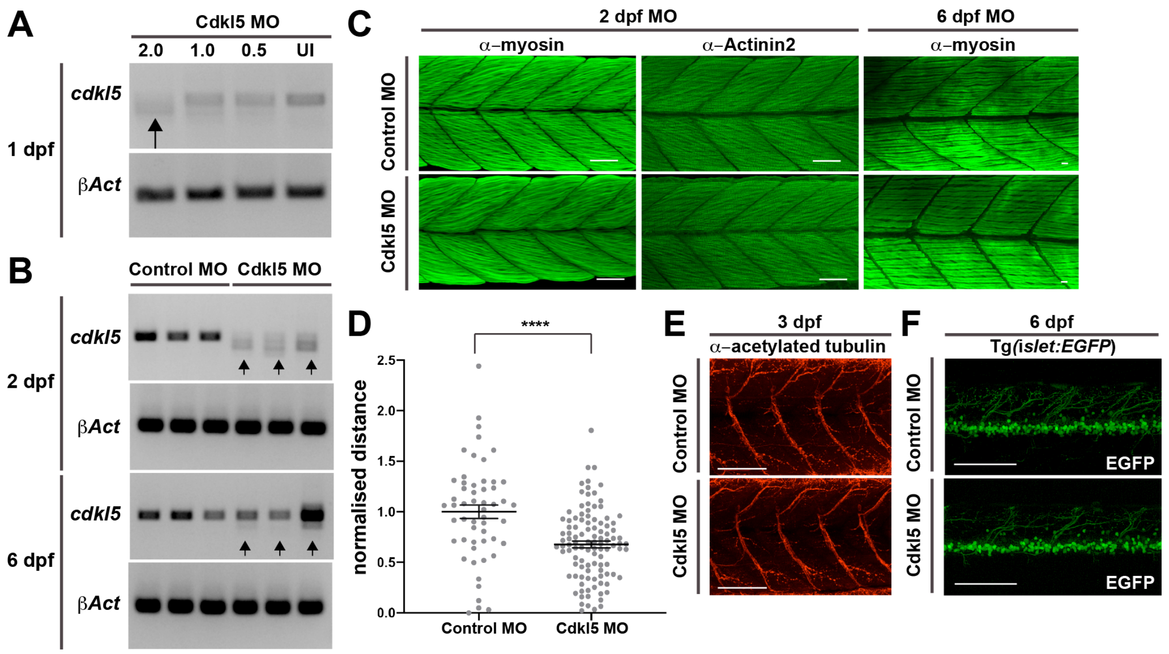

Fig. S4. Morpholino knockdown of Cdkl5. A) RT-PCR analysis for *cdkl5* mRNA at 1dpf following Cdkl5 MO injection. The amplicon in uninjected fish is the expected product size of 424 bp and is present with decreasing intensity in the Cdkl5 MO-injected fish as the injected concentration of MO increases from 0.5 ng to 2.0 ng. The lower bands (arrow) appear in the Cdkl5 MO-injected fish at the 2.0 ng concentration and results from mis-splicing of the *cdkl5* mRNA. *β-Act* was amplified as a positive control. B) RT-PCR analysis for three independent replicates at 2 dpf and 6 dpf. Mis-spliced *cdkl5* mRNA is observed in Cdkl5 MO-injected fish and not Standard Control MO-injected fish at 2 dpf. At 6 dpf, the correct *cdkl5* amplicon is present in Cdkl5 MO-injected fish, however, it is diminished in intensity, demonstrating a reduction in *cdkl5* mRNA levels compared to Standard Control MO-injected fish. *β-Act* was amplified as a positive control for each sample. C) Maximum intensity projections of confocal images of α-myosin and α-Actinin2 antibody staining at 2 dpf and α-myosin antibody staining at 6 dpf of Cdkl5 MO and Standard Control MO-injected fish. D) Quantification of normalised distance travelled of Cdkl5 MO-injected and Standard Control MO-injected fish at 6 dpf. Error bars represent mean±SEM for three independent experiments (n=18, 17, 19 Standard Control MO-injected fish and n=27, 39, 40 Cdkl5 MO-injected fish), ****$p<0.0001$, using a two-tailed t-test. E&F) Maximum intensity projections of confocal images of E) α-acetylated tubulin antibody staining of Cdkl5 MO and Standard Control MO-injected fish at 3 dpf and F) EGFP- labelled motor neurons in the spinal cord of Tg(*islet1:EGFP*) injected with either a Cdkl5 MO or Standard Control MO at 6 dpf. Scale bar = 100μm.

Disease Models & Mechanisms • Supplementary information
